# Epoxy (Meth)acrylate-Based Thermally and UV Initiated Curable Coating Systems

**DOI:** 10.3390/polym15244664

**Published:** 2023-12-11

**Authors:** Paulina Bednarczyk, Konrad Walkowiak, Izabela Irska

**Affiliations:** 1 Department of Chemical Organic Technology and Polymeric Materials, Faculty of Chemical Technology and Engineering, West Pomeranian University of Technology in Szczecin, Piastów 42 Avenue, 71-065 Szczecin, Poland; 2 Department of Materials Technology, Faculty of Mechanical Engineering and Mechatronics, West Pomeranian University of Technology in Szczecin, Piastów 19 Avenue, 70-310 Szczecin, Poland; wk42388@zut.edu.pl (K.W.);

**Keywords:** epoxy methacrylates, diglicydyl ethers, coatings, radical and cationic photopolymerization

## Abstract

Recently, photocurable coatings are being used frequently. However, it is worth mentioning that the use of photopolymerization has its drawbacks, especially in the case of curing coatings on three-dimensional surfaces and in places that are difficult to access for UV radiation. However, it is possible to develop a system in which UV technology and thermal methods for curing coatings can be combined. Moreover, the obtained resins are derived from low-viscosity epoxy resins or diglycidyl ethers, making them an ideal building material for photopolymerization-based three-dimensional printing techniques. Due to the need to improve this method, a series of epoxy (meth)acrylates containing both epoxy and (meth)acrylate groups were obtained via the addition of acrylic or methacrylic acid to epoxy resin, diglycydylether of bisphenol A epoxy resin (DGEBA), cyclohexane dimethanol diglycidyl ether (CHDMDE) and neopentyl glycol diglycidyl ether (NPDE). The structures of the synthesized copolymers were confirmed through spectroscopic analysis (FTIR) and studied regarding their nonvolatile matter content (NV) and acid values (PAVs), as well as their epoxy equivalent values (EEs). Due to the presence of both epoxy and double carbon–carbon pendant groups, two distinct mechanisms can be applied: cationic and radical. Hence, the obtained resins can be cured using UV radiation with thermally appropriate conditions and initiators. This type of method can be used as a solution to many problems currently encountered in using UV technology, such as failure to cure coatings in underexposed areas as well as deformation of coatings. Synthesized epoxy (meth)acrylate prepolymers were employed to formulate photocurable coating compositions. Furthermore, the curing process and properties of cured coatings were investigated regarding some structural factors and parameters. Among the synthesized materials, the most promising are those based on epoxy resin, characterized by their high glass transition temperature values and satisfactory functional properties.

## 1. Introduction

Photopolymerization is gaining increasing interest in the production of various types of polymeric materials with specific properties [1,2,3]. This is due to its numerous advantages, such as low energy consumption, high reaction efficiency, the ability to use solvent-free polymerizing mixtures and its minimal environmental impact [4]. For this reason, photochemically initiated radical polymerization processes play a key role in developing innovative adhesives, varnishes, photo-curable coatings, 3D printing resins, printing inks, hydrogel dental fillings and many other products [5,6,7,8]. Moreover, lately, UV-cured coatings play a crucial role in the automotive industry [9]. An alternative photopolymerization mechanism involves cationic polymerization. Cationic photopolymerization is characterized by its lower shrinkage and internal stresses compared to radical photopolymerization [10,11,12,13,14]. Materials cured by cationic photopolymerization often exhibit superior resistance to abrasion and chemicals, as well as better adhesion properties on various substrates than materials cured by radical photopolymerization [10,15,16]. Furthermore, unlike radical photopolymerization, cationic photopolymerization continues even after the light source is removed [17,18,19]. The latter, so-called the “dark reaction” method, enables more efficient monomer conversion [4,10].

Apart from utilizing photopolymerization, thermal processes can also successfully cure resins. This method is commonly used in industry to produce parts for cars or electronics. Despite its effectiveness, this technique has significant drawbacks, the most important of which is the potlife of resin formulations after mixing [20]. Furthermore, thermal curing often requires a sufficiently large oven to fit the entire sample, which can be cumbersome and impractical in some applications. Also, one can find curing in the oven to be problematic due to the high energy consumption involved; moreover, this process can be time-consuming. On the other hand, photo-curing can only be used for thin coatings because the light required to start the curing process has a very limited penetration depth, while thermal curing does not suffer from this limitation.

Traditional resins used in industry can only undergo radical or cationic polymerization. For example, (meth)acrylates and unsaturated polyesters undergo only radical polymerization, while epoxy monomers, oxetanes, and vinyl ethers follow cationic polymerization [21]. The solution to this difficulty is epoxy acrylates, the so-called vinyl ester resins (VERs), which are particularly desirable for applications such as UV-curable paints and varnishes [21]. This type of resin can be cured by both types of polymerization, which is possible due to the presence of epoxy and vinyl end groups [22]. Epoxy acrylates exhibit an exceptional combination of advantageous characteristics, encompassing flexibility, hardness, thermal resilience and resistance to yellowing [7]. These characteristics result from the epoxy backbone, which enhances its strength and flexibility [23]. Moreover, the presence of carbon–carbon and ether bonds also increases the chemical resistance of epoxy acrylates. The first commercially available VER was produced by Shell Chemical Co. in 1965, under the trade name Epocryl^®^; after that, Dow Chemical Co. introduced a similar series of resins under the name Derakane^®^ [24].

Our research is focused on VER based on monomers from petrochemical resources, which are one of the most popular building blocks for high-performance polymers such as coatings, adhesives or composites. Coatings based on such resins are relatively easy to cure and exhibit very good utilitarian properties, good adhesion, high mechanical performance, outstanding chemical resistance and low discoloration [25,26]. Currently, there are a number of analogous solutions based on monomers derived from renewable feedstocks [25]; however, there is still a strong demand for coatings with properties obtainable only for materials based on non-renewable resources.

We managed to develop a system in which it is possible to combine UV technology and thermal methods for curing coatings, which helps to exclude the use of UV technology on three-dimensional surfaces, which are difficult to expose the entire coating to because this causes their under-curing and deformation. Therefore, a series of epoxy arylates containing both epoxy and (meth)acrylate groups were obtained via the addition of acrylic or methacrylic acid to epoxy resin, bisphenol A diglycidyl ether (DGEBA), cyclohexanedimethanol diglycidyl ether (CHDMDE) and neopentyl glycol diglycidyl ether (NPDE). The chemical composition of the pre-polymers was studied employing Fourier transform infrared spectroscopy (FTIR), along with quantitative analysis encompassing non-volatile matter content (NV), partial acid values (PAVs) and epoxy equivalent (EE). The synthesized resins were thoroughly examined as formulations with either a cationic or radical photoinitiator, employing a dual-curing approach involving both UV and thermal activation methods. The obtained coatings were subjected to analysis of the curing process characteristics using the FTIR method and tests of thermal properties determined by the DSC method. The properties of the cured coating, such as hardness, gloss, adhesion, scratchability, were also tested. This systematic investigation sheds light on the optimal combinations and formulations that can lead to coatings with superior performance characteristics in various applications. Moreover, the obtained resins are derived from low-viscosity epoxy resins or diglycidyl ethers, making them an ideal building material for photopolymerization-based three-dimensional printing techniques [27,28]. This applicability is further demonstrated by the dual-curing method, which first employs UV light, and then thermal curing. Conventional goods produced by 3D printing photopolymer resins are post-cured in UV chambers [29]. The research carried out in this study demonstrates that VER’s curing process effectively replicates the 3D printing workflow, where LED-initiated curing is followed by post-curing. However, the latter should be conducted in an oven at an elevated temperature instead of in an irradiation chamber. These advancements highlight the potential of our newly developed VERs in the realm of additive manufacturing.

## 2. Materials and Methods

### 2.1. Materials

The following resins were used: the epoxy resin Epidian 6^®^ (Ep6), Organika-Sarzyna S.A. (Nowa Sarzyna, Poland), with the epoxide number 185–196 g/mol and viscosity 700–1100 mPa·s at 25 °C; bisphenol A glycidyl ether (DGEBA), Sigma–Aldrich (Gillingham, Dorset, UK), with the epoxide equivalent of 171.4 g/mol and viscosity of 5032 mPa·s at 25 °C; cyclohexanedimethanol diglycidyl ether (CHDMDE) under the trade name Grilonit^®^ V 51-63, EMS-GRILTECH (Domat/Ems, Switzerland), with an epoxide equivalent of 158.8 g/mol and viscosity of 70 mPa·s at 25 °C; and neopentyl glycol diglycidyl ether (NPDE), Tokyo Chemical Industry (Tokyo, Japan), characterized by the epoxide equivalent of 146.8 g/mol and viscosity of 10 mPa·s at 25 °C. Furthermore, acrylic acid (AA) and methacrylic acid (MAA) supplied by Acros Organics (Geel, Belgium) with a purity of 99.5% were used for the synthesis of epoxy arylates. The chemical structure of the used resins can be found in Table 1. The catalyst used to carry out the reaction was triphenylphosphine (PPh_3_) Apollo Scientific (Bredbury, UK), while hydroquinone (HQ) Acros Organics (Geel, Belgium) was employed as a polymerization inhibitor. 

For titration, the following reagents and indicators were used: glacial acetic acid, toluene, potassium hydroxide standard solution 0.1 M in ethanol (KOH) and crystal violet purchased from Chempur (Piekary Slaskie, Poland); chloroform from P.P.H. Stanlab (Lublin, Poland); ethyl alcohol from Avantor (Gliwice, Poland); tetraethylammonium bromide provided by Acros Organics (Geel, Belgium); perchloric acid standard solution 0.1 M in glacial acetic acid supplied by Fischer Chemicals (Zurich, Switzerland); and phenolophtalein 1% in ethyl alcohol solution from Eurochem BGD (Tarnów, Poland). All of the chemicals were analytical grade. 

The radical photoinitiator utilized in this study was ethyl-2,4,6-trimethylbenzoylphenylphosphinate (Lucirin TPO-L) purchased from BASF (Ludwigshafen, Germany). As the cationic photoinitiator, a mixture of triarylsulfonium hexafluorophosphate salts (Cyracure UV6992) Dow (Frankfurt, Germany) was employed. Furthermore, as a thermal initiator for cationic polymerization, triethylenetetramine (Z-1) CIECH S.A. (Warsaw, Poland) was used, and for radical polymerization, benzoyl peroxide (Sigma-Aldrich, Gillingham, Dorset, UK).

### 2.2. Synthesis of Epoxy (Meth)acrylate Resins

Due to the fact that this research is a coherent continuation of our previous research on the course of synthesis of various epoxy (meth)acrylates, the detailed procedure regarding the synthesis conditions is known [7,21,26]. However, in general, the synthesis of epoxy (meth)acrylate resins was carried out in a 250 mL, three-neck glass reactor. The reactor is equipped with a thermometer, condenser, nitrogen inlet and mechanical stirrer. At the beginning, the chosen diglycidyl ether was incorporated into the reactor, followed by the addition of HQ (0.0075 wt.% on total batch weight). Then, the AA or MAA (0.5 mol in relation to resin epoxy value) and the catalyst PPh_3_ (0.8 wt.% in relation to the mass of added acid) were added. The mixture was heated up to 70 °C using an oil bath and stirred (120 RPM) to obtain a homogenous mixture. Subsequently, the temperature was set to 90 °C, and the process was conducted for 4–6 h in a nitrogen atmosphere and constantly stirred (120 RPM). The prepared EAs appear as colorless, transparent, viscous liquids. The synthesized EAs were coded as Ep6-AA, Ep6-MAA, DGEBA-AA, DGEBA-MAA, NDPE-AA, NDPE-MAA, CHDMDE-AA and CHDMDE-MAA.

### 2.3. Preparation of Coating Formulations and Cured Films

The coating formulations were formulated using the synthesized EAs. In both the UV-curing and hybrid curing formulations, a 3% by weight addition of photoinitiators relative to the resin content was incorporated. The amount of thermal cationic initiator was calculated and added according to the amount of epoxy equivalent of each resin. 

The components were stirred under dark conditions until a homogeneous mixture was achieved. The formulations were applied on glass substrates using a gap applicator (120 μm). The UV curing of the polymeric film was carried out under a light source (UV lamp, Aktiprint-mini 18-2, type: UN50029, Technigraf GmbH, Gravenwiesbach, Germany) at room temperature and irradiated under UV light with an intensity of 200 mW/cm^2^ to dryness. The thermal curing of the polymeric film was performed in a dryer set at 80 °C. In the dual-curing process, the UV-curing step was performed prior to the thermal-curing step. The polymerization method with cationic and radical initiators was coded as UV(R)-T(C) and with the use of both cation initiators as UV(C)-T(C). 

### 2.4. Characterization Methods

Fourier transform infrared spectra (FTIR) were obtained using a Nicolet iS 5 FTIR spectrometer (Thermo Fisher Scientific Inc., Waltham, MA, USA) with 16 scans over the 4000–400 cm^–1^ frequency range.

Determining the non-volatile matter content (NV) was conducted through thermogravimetric analysis using a moisture analyzer (MAX 60/NP, Radwag, Radom, Poland), following the guidelines outlined in the ISO 3251:2019 standard. Consequently, the partial acid values (PAVs) and epoxy equivalents (EEs) were analyzed using colorimetric titration according to EN ISO 2114:2000 and EN ISO 3001:1999. The procedure is described in more detail in previous studies [7,21,26].

The DSC method was used to determine the thermally reversible mechanism of the DA-structures in the cured coatings. The study used a DSC apparatus (Q100, TA Instruments, New Castle, DE, USA). Aluminum pans were utilized to seal samples weighing between 7 and 10 mg. With a heating ramp of 10 °C/min, measurements were conducted in a heating–cooling–heating cycle in a temperature range of −50 to 190 °C.

The following tests were conducted to study the properties of the cured coatings: the pendulum hardness, scratchability, cross-cut adhesion and gloss tests. The hardness of the coatings was analyzed using a Persoz pendulum on a glass substrate (TQC Sheen, Capelle aan den Ijssel, The Netherlands) according to the ISO 1522 standard. Scrachability was analyzed using a sclerometer hardness tester, a professional type scratch resistance tester (TQC Sheen, Capelle aan den IJssel, The Netherlands), according to the ISO 1518 standard. The cross-cut adhesion test was performed according to the PN-EN ISO 2409 (using equipment from BYK, Wesel, Germany). The gloss was measured with a spectrometer GLS (SADT Development Technology Co., Ltd., Beijing, China) following the guideline of ASTM D523. The substrate used for the tests was a glass plate.

## 3. Results

### 3.1. Synthesis Parameters and Properties of Obtained Epoxy (Meth)acrylates

During the synthesis, the samples were collected and subjected to FTIR and titration (PAVs and EE); after synthesis, NV analysis was performed. The objective was to control the progression of the synthesis process and, through prior synthesis, to determine the optimum temperature and synthesis time. By monitoring PAVs and EE, a dependence between synthesis time and the percentage of reacted epoxide (EGC) and AA or MAA (AAC or MAAC) was determined, as shown in Table 2. One can observe a significant shift in the mentioned values after the 120-min mark. This phenomenon is attributed to the high concentration of reactive sites during the initial stage. As a result, the reaction between the acid and epoxide proceeds easily. However, as the reaction progresses, the decreasing availability of reactive sites progressively hinders the overall process [21]. In the final stage, the EGC for most of the studied resins was around 50%. A significant difference was observed for CHMEDE-AA, whose value of EGC was approximately 31%. The difference in results can be the result of distinct chain architecture, reactivity, viscosity, molecular weight and molecular mobility, which was also observed in one of our recent studies [30]. Therefore, it is impossible to state any single factor that determines the rate of response, as well as the degree of conversion of EG and AA or MAA (Table 2).

### 3.2. Characteristics of the Dual-Curing Process of the Epoxy (Meth)acrylates

In our previous studies [7,21,26,30] we described in detail the dual-curing process of epoxy (meth)acrylate (EA) coatings, specifically the use of two different photopolymerization mechanisms, i.e., radical and cationic. In turn, this study presents a dual-curing process that takes into account various initiation methods, i.e., using electromagnetic radiation and elevated temperature. The combination of two methods for initiating the polymerization process is of great practical importance when curing coatings on three-dimensional surfaces, in particular for the so-called “shadow” places, i.e., places where UV radiation does not reach a problem results with insufficient curing of the coating. For this purpose, several curing methods were compared: (i) UV(C)—photocuring of the EA composition containing a cationic photoinitiator; (ii) UV(R)—photocuring of the EA composition containing a radical photoinitiator; (iii) T(C)—thermal curing of the EA composition containing a thermal initiator of the cationic process; (iv) UV©(C)—two-stage dual-curing process, cationic photocuring, and then thermal-curing process in the cationic process; and (v) UV(R)-T(C)—two-stage dual-curing process, radical photocuring, and then thermal-curing process in the cationic process. To sum up, methods (i)–(iii) run as a one-stage process, while methods (iv)–(v) run as a two-stage process. Moreover, methods (i), (iii), (iv—part one and two) and (v—part two) involve cationic polymerization of epoxy groups with the formation of a polyether network, while methods (ii) and (v—part one) involve radical polymerization of groups (meth)acrylate (acrylate or methacrylate) with the formation of a polyester network. There were also tests of thermal curing according to the radical mechanism using peroxide initiators, but unfortunately, they ended in failure; hence, this type of solution was omitted in further analysis.

Using FTIR spectroscopy, the curability of the films was investigated in terms of the type of curing mechanism and the source of curing. Comparative FTIR spectra of the uncured formulations and cured films are illustrated in Table 3. The analysis centered on specific absorption bands: epoxy ring stretching vibration (-C-O) at 915 cm^−1^ and C-O-C (~825 cm^−1^) stretching vibrations [31,32]; ether groups (-C-O-C) at 1036 cm^−1^ [33]; and carbonyl stretching mode (-C=C) from 1607 cm^−1^ to 1635 cm^−1^ [34,35] and 809 cm^−1^ [30] associated with acrylic or methacrylic acid. Moreover, the absorption peak corresponding to C=O stretching vibrations was observed at 1718 cm^−1^. Photocuring with a cationic initiator resulted in a reduced absorption band at 915 cm^−1^, occasionally disappearing entirely, indicating successful resin curing through this method. Similarly, photocuring with a radical photoinitiator led to a decrease in intensity of the characteristic band spanning from 1607 cm^−1^ to 1635 cm^−1^ and the peak at 809 cm^−1^, alongside the persistence of epoxy groups; this suggests that polymerization via unsaturated bonds originating from double bonds occurred [26]. Thermal curing utilizing a cationic initiator reflected the outcomes of photocuring with a cationic initiator. In the case of hybrid curing using only a cationic initiator, the spectra at 915 cm^−1^ almost completely faded out as a consequence of cationic ring-opening polymerization. Moreover, the hybrid curing that involved the radical photoinitiator and cationic thermal initiator was successful as evidenced by significant decreases in peaks at 915 cm^−1^ and those associated with carbonyl stretching mode. The broadband that spans from 3200 cm^−1^ to 3600 cm^−1^ is associated with -O-H groups. 

**Figure 1:**
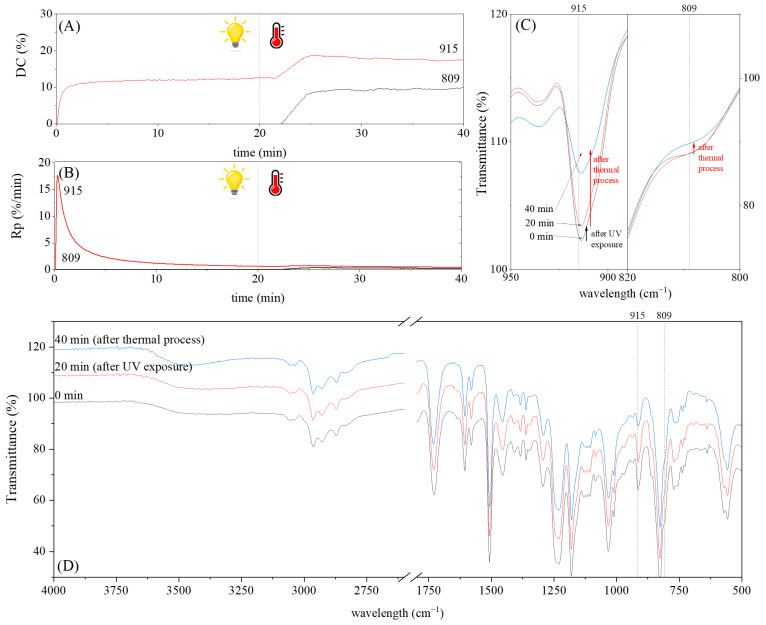
The UV(C)-T(C) dual-curing process of EA monitored via FTIR including (**A**) change in the degree of conversion of epoxy groups (DC) and (**B**) polymerization rate (Rp) as well as (**C**) change in the height of selected peaks and (**D**) full spectra after 20 and 40 min of curing.

**Figure 2:**
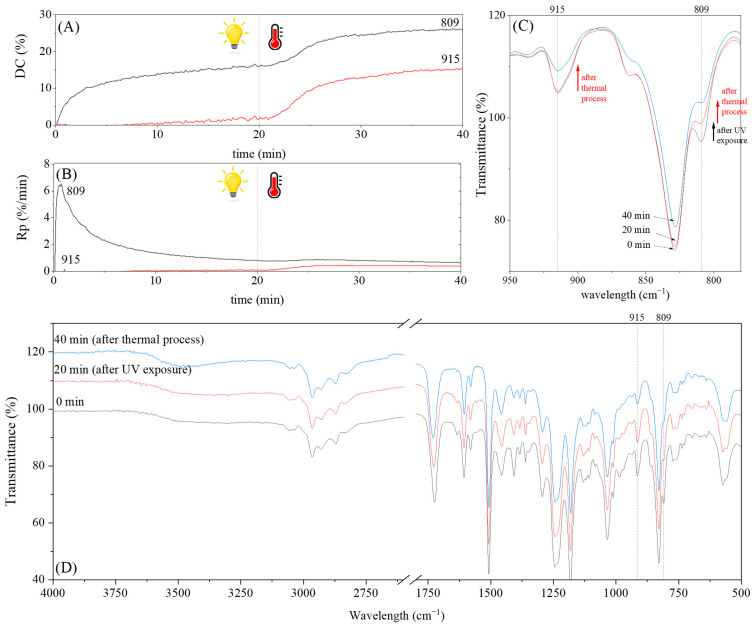
The UV(R)-T(C) dual-curing process of EA monitored via FTIR including (**A**) change in the degree of conversion of acrylate groups (DC) and (**B**) polymerization rate (Rp) as well as (**C**) change in the height of selected peaks and (**D**) full spectra after 20 and 40 min of curing.

**Figure 3:**
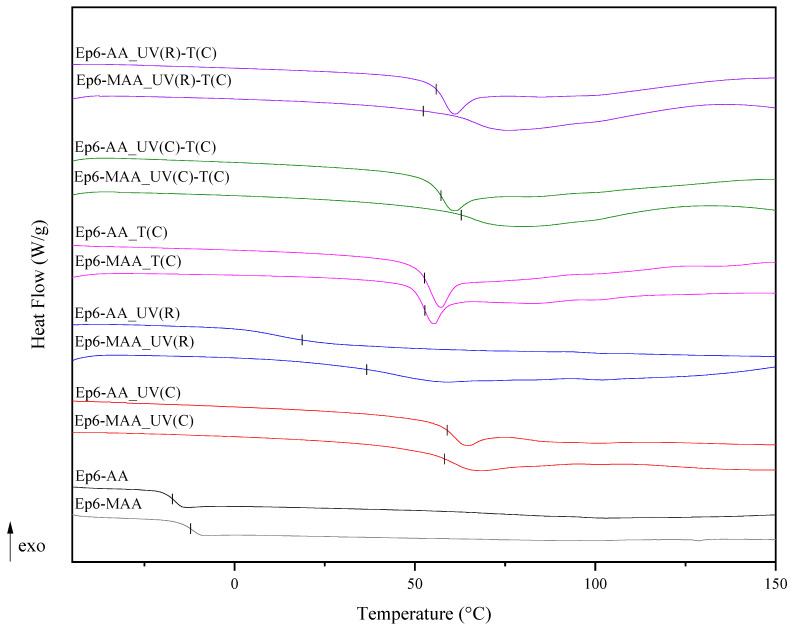
Curing process of Ep-6-based compositions monitored via FTIR.

The dual method of EA curing, i.e., a combination of photocuring and thermal curing, was analyzed in detail based on the example of the Ep6-MAA resin. The first stage lasted 20 min and was divided into cationic polymerization of epoxy groups and radical polymerization of (meth)acrylate groups. The second stage also lasted 20 min, and was a cationic process with the cationic thermal initiator. Photopolymerization according to two different mechanisms allows distinguishing EA in terms of the formation of polyether or polyether networks, which is reflected in the curing rate, conversion of functional groups and ultimately the thermal properties or utilitarian properties of the cured coatings. Figure 1 and Figure 2 show the degree of conversion of epoxy or (meth)acrylate groups depending on the polymerization mechanism (DC, %), polymerization rate (Rp, %/min) and changes in the characteristic peaks present in the FTIR spectrum; in particular, the 915 cm^−1^ peak is attributed to epoxy ring stretching vibration (-C-O) and the course of cationic polymerization, and 809 cm^−1^ is attributed to carbonyl stretching mode (-C=C) and radical polymerization. Figure 2 shows the monitoring results regarding the cationic process; therefore, in the case of the 809 cm^−1^ peak, no significant changes were observed. In turn, the peak corresponding to epoxy groups decreases its intensity slightly in the photopolymerization process; this is reflected in the over 10% conversion obtained; then, this change progresses in the thermal process to obtain less than 20% conversion of the epoxy groups, and the coating can be considered cured. Figure 3 shows the results related to the monitoring of the radical process; therefore, in this case, the 915 cm^−1^ peak does not show significant changes, while the peak responsible for the unsaturated bonds of (meth)acrylate groups is reduced, which is reflected in the approximately 15% conversion achieved of these groups. In turn, as in the previous case, both processes undergo thermal curing, which is probably related to the formation of a polyether–polyester network.

### 3.3. The Thermal Properties of the Obtained EAs

The thermal properties of the cross-linked EA networks are greatly influenced by the structure, chemical composition, type and concentration of remaining polar groups, cohesive energy between molecular chains, molecular chain rigidity, different interaction parameters and other chemical structural factors like sferic strain, conformational arrangements of groups, etc. [23]. The DSC analyses of the obtained EA resins and the coatings cured using various methods were carried out. The representative spectra are shown in Figure 3. The corresponding data on the glass transition temperature are tabulated in Table 4. It was observed that the curing method had a large impact on the glass transition temperature of the polymers. It is clearly visible that the one-stage curing process using UV radiation and a radical photoinitiator led to a lower glass transition temperature than in the case of other methods. This was probably due to the formation of different polymer networks, i.e., the polyester or polyether network, the degree of their cross-linking or the ordering of the polymer chains. Furthermore, this fact may be also attributed to the decrease in the macromolecular mobility as a result of imposing restrictions on the ordering phenomenon inside the polymer due to an increase in the crosslinking density. The T_g_ was correspondingly higher for the EA-based coatings with a rigid carbon chain structure, i.e., lower segmental mobility, such as those based on Ep6 and DGEBA containing aromatic groups, and for methacrylates compared to acrylates due to the presence of an additional methyl group. Hence, the T_g_ for Ep6-MAA was 37 °C for the radical process, and for the cationic or dual processes it was in the range of 53–63 °C; the T_g_ for Ep6-AA was 19 °C for the radical process, and for the cationic or dual processes it was in the range 52–59 °C; and, in turn, the T_g_ for CHDMDE-AA was only 2 °C for the radical process, and for the cationic or dual processes, it was in the range of 3–25 °C. The values of the glass transition temperatures of the resins and compositions were higher than the T_g_ of EBECRYL^®^ 860 (13 °C) [36], and almost equal to or lower than that for furfuryl methacrylate/styrene with wt.% of FM (78 °C) [37]. 

### 3.4. Properties of Cured Films

The influence of various factors on the properties of the cured coatings was studied. These factors were the following: the type of polymerization (cation or radical), the source of curing, different types of resins and the addition of acrylic or methacrylic acid (Table 5). The coatings’ performance was analyzed to deepen the understating of each parameter on properties like hardness, scratchability, adhesion and gloss. In general, the highest hardness values for most resins were observed during cationic polymerization with thermal curing. This was especially observed for the formulation based on Ep6 and DGEBA, as only their hardness notably surpassed 300. However, thermal radical polymerization could not be achieved, and certain formulations did not undergo curing through thermal cationic polymerization. The scratchability, adhesion, and gloss tests resulted in higher values in the case of cationic photopolymerization when compared to radical photopolymerization. In the hybrid system of curing, one can observe that UV(C) + T(C) and UV(R) + T(C) depend on the composition. The best properties exhibited were from the cured films based on Ep6 and DGEBA, which underwent UV(R) + T(C) polymerization (except DGEBA + AA). Notably, the difference in hardness between the DGEBA–MMA film subjected to the UV(R) + T(C) polymerization mechanism and the film treated with the UV(C) + T(C) polymerization mechanism is about 84 s. The properties like scratchability, adhesion and gloss are similar for cured films that underwent UV(C) + T(C) and UV(R) + T(C), with a slight advantage for UV(R) + T(C). When comparing the presence of acrylate and methacrylate groups, the overall formulation that contains methacrylate groups has greater hardness. This is possible due to the greater rigidity of polymer chains containing methacrylate groups, which was also observed by [21,38]. Among the studied methods and formulations, the best properties are exhibited by a formulation based on Ep6 and DGEBA. The exceptional performance of this formulation can be attributed to the unique chemical structure of these resins. Unlike the other tested resins, Ep6 and DGEBA possess a more rigid chemical structure in comparison to other tested resins due to the presence of a benzene ring, which hinders the movement of particles.

## 4. Conclusions

The obtained epoxy (meth)acrylate prepolymers successfully underwent the curing process that used both UV and thermal methods. Hence, they can be used as coatings for three-dimensional surfaces that are difficult to access for UV radiation, i.e., the so-called “places of shadow”. The influence of various factors on the properties of the cured coatings was studied. These factors were the following: the type of polymerization (cation or radical), the source of curing, different types of resins and the addition of acrylic or methacrylic acid. As a result of using various methods and curing mechanisms, coatings with different thermal properties were obtained. Hence, it can be concluded that various types of polymer networks can be formed, which determine the properties of the coatings. The coatings’ performance was also analyzed to deepen the understating of each parameter on properties like hardness, scratchability, adhesion and gloss. Coatings with the best properties were obtained from compositions based on Ep6. They have the highest or one of the highest T_g_ values before and after curing. The compositions based on Ep6 exhibit among the most exceptional values of pendulum hardness, adhesion and gloss, irrespective of the employed curing method. In this case, it is difficult to find a universal rule to select the curing method for the best properties of the cured coatings. However, it can be generally stated that the obtained coatings have a wide range of properties, which makes them suitable for use as various types of coating materials.

## Figures and Tables

**Table 1:** Chemical structures of the used resins.

Resin	Chemical Structure
Epidian 6^®^ (Ep6)	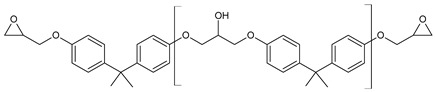
Bisphenol A glycidyl ether (DGEBA)	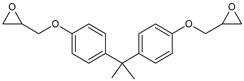
Cyclohexanedimethanol diglycidyl ether (CHDMDE)	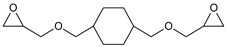
Neopentyl glycol diglycidyl ether (NPDE)	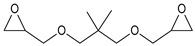

**Table 2:** The degrees of conversion of EG and AA or MAA of investigated epoxy (meth)acrylates.

	AA	MAA
Ep6	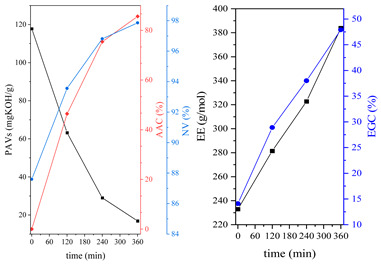	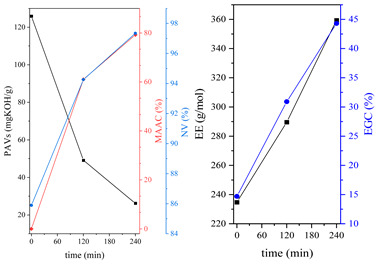
DGEBA	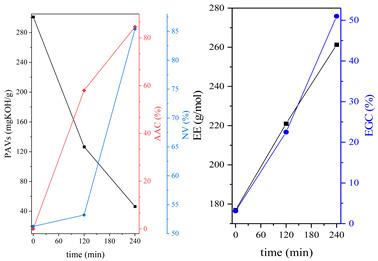	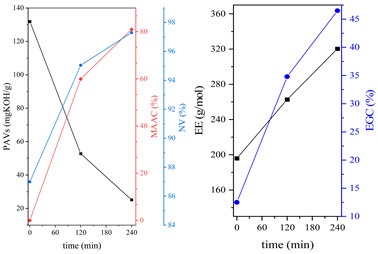
NPDE	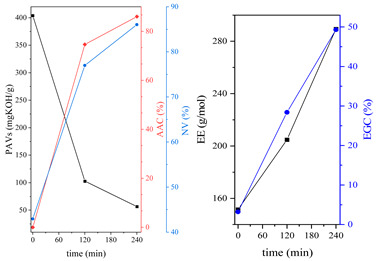	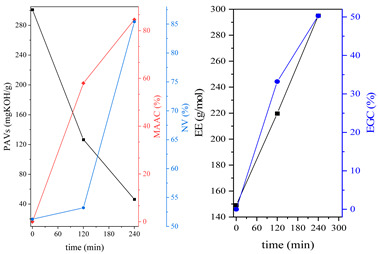
CHDMDE	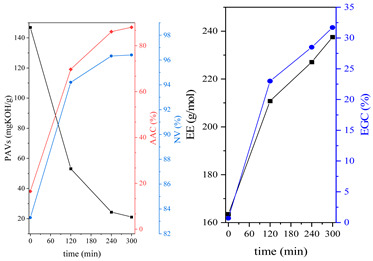	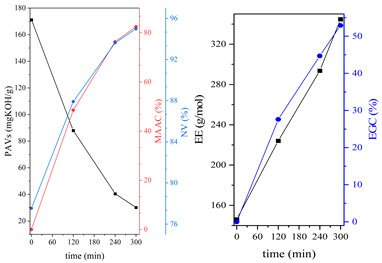

**Table 3:** The FTIR spectra of investigated epoxy (meth)acrylates depending on the curing method.

	AA	MAA
Ep6	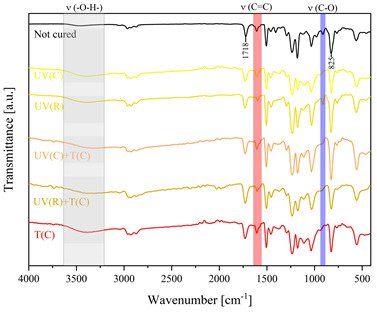	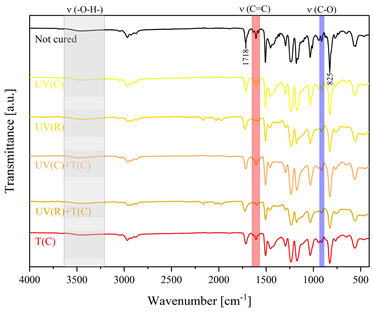
DGEBA	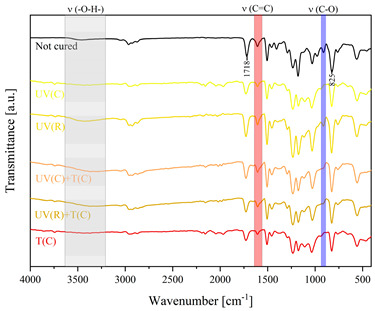	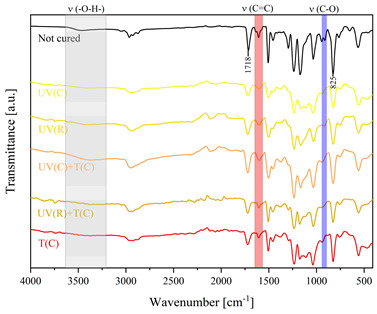
NPDE	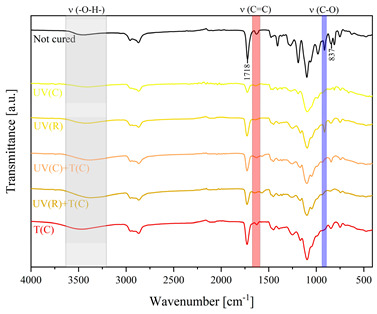	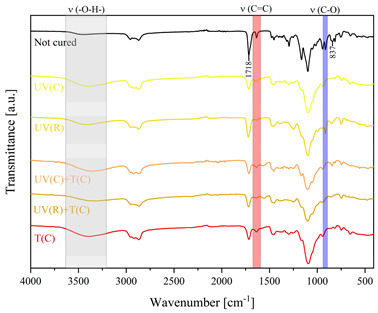
CHDMDE	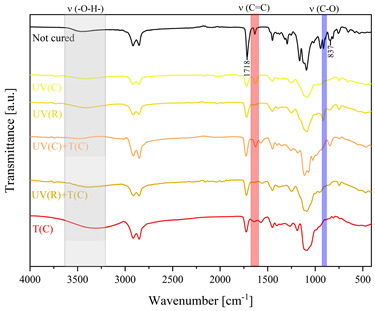	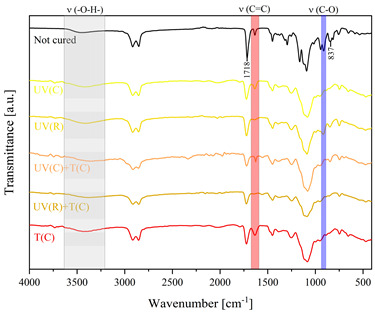

**Table 4:** Glass transition temperature (T_g_, °C) data of EAs in liquid and cured form using various methods.

Sample	No Cure	UV(C)	UV(R)	T(C)	UV(C) + T(C)	UV(R) + T(C)
Ep6-MMA	−12	58	37	53	63	62
Ep6-AA	−17	59	19	52	57	56
DGEBA-MAA	−15	53	39	41	60	58
DGEBA-AA	−13	60	28	52	56	57
NDPE MAA	−39	42	30	-	56	53
NDPE AA	−45	11	9	10	19	21
CHDMDE-MAA	−48	31	14	-	31	46
CHDMDE-AA	−43	21	2	3	18	25

**Table 5:** The properties of the cured coatings with the application of UV, thermal initiators and dual curing (UV/thermal).

Sample	Hardness		Scratchability (N)		Adhesion		Gloss (GU)	
UV curing	UV(C)	UV(R)	UV(C)	UV(R)	UV(C)	UV(R)	UV(C)	UV(R)
Ep6-MMA	299 ± 3	117 ± 2	1 ± 0.5	1 ± 0.25	5	1	444 ± 6	168 ± 5
Ep6-AA	303 ± 3	58 ± 2	1 ± 0.5	0.2 ± 0.25	4	0	467 ± 5	113 ± 2
DGEBA-MAA	300 ± 3	138 ± 2	1 ± 0.25	1.5 ± 0.25	5	1	400 ± 5	145 ± 3
DGEBA-AA	278 ± 3	90 ± 2	1 ± 0.25	1 ± 0.25	5	1	450 ± 5	139 ± 5
NDPE-MAA	51 ± 2	42 ± 1	0.5 ± 0.25	0.2 ± 0.25	0	1	80 ± 2	144 ± 2
NDPE AA	26 ± 1	78 ± 2	0.5 ± 0.25	0.2 ± 0.25	0	1	18 ± 5	124 ± 2
CHDMDE-MAA	103 ± 2	43 ± 1	0.5 ± 0.25	1.5 ± 0.25	3	1	9 ± 3	122 ± 2
CHDMDE-AA	122 ± 2	64 ± 2	1.5 ± 0.25	0.5 ± 0.25	1	0	102 ± 2	122 ± 2
Thermal curing	T(C)	T(R)	T(C)	T(R)	T(C)	T(R)	T(C)	T(R)
Ep6-MMA	351 ± 3	-	1 ± 0.25	-	1	-	156 ± 3	-
Ep6-AA	359 ± 3	-	1 ± 0.25	-	1	-	205 ± 5	-
DGEBA-MAA	320 ± 3	-	1.5 ± 0.25	-	1.5	-	110 ± 3	-
DGEBA-AA	352 ± 3	-	1 ± 0.25	-	1	-	210 ± 3	-
NDPE MAA	-	-	-	-	-	-	-	-
NDPE AA	42 ± 2	-	0.5 ± 0.25	-	0.5	-	200 ± 3	-
CHDMDE-MAA	-	-	-	-	-	-	-	-
CHDMDE-AA	46 ± 2	-	1 ± 0.25	-	1	-	300 ± 3	-
Dual curing	UV(C) + T(C)	UV(R) + T(C)	UV(C) + T(C)	UV(R) + T(C)	UV(C) + T(C)	UV(R) + T(C)	UV(C) + T(C)	UV(R) + T(C)
Ep6-MMA	226 ± 3	266 ± 3	2.5 ± 0.5	2.5 ± 0.25	3	5	185 ± 4	208 ± 3
Ep6-AA	231 ± 3	277 ± 3	1.5 ± 0.25	1.5 ± 0.25	3	3	195 ± 3	211 ± 2
DGEBA-MAA	250 ± 3	334 ± 4	2.5 ± 0.5	5 ± 0.5	3	5	144 ± 3	145 ± 1
DGEBA-AA	238 ± 3	186 ± 2	2.5 ± 0.5	3.5 ± 0.5	0	0	193 ± 3	185 ± 4
NDPE MAA	219 ± 2	78 ± 2	6 ± 0.5	1.5 ± 0.25	3	3	103 ± 2	95 ± 2
NDPE AA	65 ± 2	31 ± 2	2.5 ± 0.25	1 ± 0.25	0	0	128 ± 3	173 ± 3
CHDMDE-MAA	88 ± 2	102 ± 3	4 ± 0.5	3.5 ± 0.25	4	4	100 ± 2	102 ± 2
CHDMDE-AA	50 ± 2	30 ± 1	2.5 ± 0.25	1.5 ± 0.25	0	0	105 ± 2	176 ± 3

## Data Availability

Data are contained within the article.
